# MiR-320 inhibits PRRSV replication by targeting PRRSV ORF6 and porcine CEBPB

**DOI:** 10.1186/s13567-024-01309-7

**Published:** 2024-05-15

**Authors:** Xiaoxiao Gao, Xiangbin You, Guowei Wang, Mengtian Liu, Longlong Ye, Yufeng Meng, Gan Luo, Dequan Xu, Min Liu

**Affiliations:** 1 Colleges of Animal Science and Technology/College of Veterinary Medicine, Huazhong Agricultural University, Wuhan, 430070 China; 2 Key Laboratory of Swine Genetics and Breeding of Ministry of Agriculture and Rural Affairs, Huazhong Agricultural University, Wuhan, 430070 China; 3 Key Laboratory of Agricultural Animal Genetics, Breeding and Reproduction of Ministry of Education, Huazhong Agricultural University, Wuhan, 430070 China; 4 College of Animal Science and Technology, Henan University of Science and Technology, Luoyang, 471023 China

**Keywords:** miR-320, CCAAT enhancer binding protein beta, PRRSV, ORF6, pig

## Abstract

Porcine reproductive and respiratory syndrome (PRRS), a highly contagious disease caused by Porcine reproductive and respiratory syndrome virus (PRRSV), results in huge economic losses to the world pig industry. MiRNAs have been reported to be involved in regulation of viral infection. In our study, miR-320 was one of 21 common differentially expressed miRNAs of Meishan, Pietrain, and Landrace pig breeds at 9-h post-infection (hpi). Bioinformatics and experiments found that PRRSV replication was inhibited by miR-320 through directly targeting PRRSV ORF6. In addition, the expression of CCAAT enhancer binding protein beta (CEBPB) was also inhibited by miR-320 by targeting the 3ʹ UTR of CEBPB, which significantly promotes PRRSV replication. Intramuscular injection of pEGFP-N1-miR-320 verified that miR-320 significantly inhibited the replication of PRRSV and alleviated the symptoms caused by PRRSV in piglets. Taken together, miR-320 have significant roles in the infection and may be promising therapeutic target for PRRS.

**Supplementary Information:**

The online version contains supplementary material available at 10.1186/s13567-024-01309-7.

## Introduction

Porcine reproductive and respiratory syndrome (PRRS) is a highly contagious disease also known as “porcine blue ear disease”, which is characterized by reproductive disorders in pregnant sows and respiratory diseases in pigs of various ages, especially in piglets [1, 2]. PRRS was first detected in Beijing, China, in 1995, followed by a massive outbreak in 2006 [3–5]. Now, various types of porcine reproductive and respiratory syndrome virus (PRRSV) strains are widespread and cause a lot of economic losses [6, 7]. The common reproductive clinical symptoms of PRRS include delayed returns to estrus, premature birth, late abortion, stillbirth, weak and mummy fetuses in sows, a lack of libido and decreased semen quality in boars.

PRRSV is a single-stranded positive sense, non-segmented RNA virus and consists of 9 open reading frames (ORFs), ORF1a, ORF1b, ORF2a, ORF2b, ORF3, ORF4, ORF5, ORF6, and ORF7 [8, 9]. ORF1a and ORF1b encode non-structural proteins related to viral replicase and RNA polymerase [10]. ORF2a, ORF2b, and ORF3 ~ ORF7 are located at the 3’ end of the viral genome and encode major structural proteins [11].

MicroRNAs (miRNAs) are a class of endogenous non-coding RNAs with regulatory function that are about 20–25 nucleotides in length [12]. MiRNAs guide RNA-induced silencing complex (RISC) to degrade the mRNA or hinder translation by base pairing with target mRNA [13]. Numerous studies have shown that miRNAs are involved in the regulation of cell growth, tissue differentiation, embryonic development and disease [14–16]. Previous reports also indicated that miRNAs are involved in PRRSV infection. Xiao et al. has shown that PRRSV replication was promoted by miR-22 by targeting the host factor HO-1 [17]. Another report has shown that PRRSV replication was inhibited by miR-23 through directly targeting PRRSV and upregulating type I interferon [18]. In addition, several studies also have shown that miR-30c, miR-181, miR-130 could be used to treat PRRSV-infected pigs in vivo [19–21].

In this study, miR-320 was found to be significantly differentially expressed in infected/mock-infected porcine alveolar macrophages (PAMs) of Meishan, Pietrain, and Landrace pig breeds. Furthermore, miR-320 was predicted to target PRRSV ORF6, which was confirmed by double fluorescence reporter assay. In addition, miR-320 also inhibited the expression of CEBPB by targeting the 3' UTR of CEBPB, which promotes PRRSV replication. And the replication of PRRSV was inhibited by intramuscular injection of pEGFP-N1-miR-320, alleviating the symptoms caused by PRRSV in piglets. Taken together, miR-320 inhibits PRRSV replication by targeting ORF6 and CEBPB.

## Materials and methods

### Ethics statement

All animal procedures were approved by the Scientific Ethic Committee of Huazhong Agricultural University, Wuhan, China (ID number: HZAUSW2015-018).

### Cell line and virus

Marc-145 cells were obtained from China Center for Type Culture Collection (CCTCC) and cultured in DMEM (Hyclone, Logan, UT, USA) supplemented with 10% fetal bovine serum (CLARK Bioscience, Virginia, USA) and maintained in an incubator at 37 ℃ with 5% CO_2_. PRRSV WuH3 strain (GenBank accession No.HM853673) was kindly provided by Dr Xiao Shaobo.

### MiRNA-seq, bioinformatics and luciferase reporter assay

PAMs were isolated from Pietrain (P), Qingping (QP), Meishan (MS), and Landrace (L) pig breeds by bronchoalveolar lavage under aseptic conditions [45]. The washed cells were incubated in 10 cm^2^ culture dishes at 37 °C and 5% CO_2_ for 2 h; the unadhered cells were removed and the remaining cells were digested and transferred to a suitable cell culture plate for subsequent experiments. Then, the PAMs of 5 pigs of each breed were infected with PRRSV strain WuH3 at multiplicity of infection (MOI) of 0.1 PFU/cell. The PRRSV-infected PAMs were collected at 9, 36, and 60 hpi and mixed evenly. The control group (mock-infected) PAMs were infected with culture medium and collected at 9, 36, and 60 h. Total cellular RNA was isolated using the Trizol reagent (Invitrogen, Cashman, CA, USA). The miRNA fragments (18–30 nt) were isolated from total RNA by polyacrylamide gel electrophoresis and 3′-adaptor (TGGAATTCTCGGGTGCCAAGG) was first ligated to the RNA 3′ ends. Then the 5′ adaptor (GTTCAGAGTTCTACAGTCCGACGATC) was ligated to 5′end of the preparation. T4 RNA Ligase (Takara, Dalian, China) was used in the ligation reaction. The adaptor-ligated miRNA was then converted to cDNA using SuperScript II Reverse Transcriptase (Life Technologies, Carlsbad, CA, USA). The resulting cDNA was amplified on the PCR machine. The purified PCR products were recovered with QIAquick Gel Extraction Kit (Qiagen, Beijing, China) following the manufacturer’s instruction and assessed on an Agilent Technologies 2100 Bioanalyzer (Agilent Technologies, Santa Clara, CA, USA). Each miRNA library was sequenced individually using Illumina/solexa Genome Analyzer (BGI, Shenzhen, China. The expression of miRNAs was normalized and analyzed by calculating fold-change and p-value [46, 47]. A miRNA was labeled as differentially expressed, when |log_2_(fold change)|≥ 1 and *p* ≤ 0.01. TargetScan, miRbase, and RNAhybrid software were used to predict the target mRNAs of miRNAs. MiR-320, which is strongly downregulated in patients with COVID-19 induced severe respiratory failure [48], is differentially expressed before and after infection with PRRSV among different pig breeds. The potential miR-320 binding sites in the genome of PRRSV WuH3 strain were predicted using ViTa and RNA hybrid software. Using porcine genome DNA as template, the 3'UTR of CEBPB and ORF6 containing putative miR-320 binding site were amplified by PCR with primers CEBPB-W-F/CEBPB-W-R and ORF6-W-F/ORF6-W-R, and cloned into pmirGLO vector (Promega, Madison, Wisconsin, USA) and designated as pmirGLO-CEBPB-WT and pmirGLO-ORF6-WT, respectively. Mutations (CGGTCT and CCGTCGT) in the miR-320 predicted target site (CCCAGT and CCCGGCT) in the 3’UTR were generated by PCR with primers CEBPB-M-F/CEBPB-M-R and ORF6-M-F/ORF6-M-R, and cloned into pmirGLO vector (Promega, Madison, Wisconsin, USA) and designated as pmirGLO-CEBPB-MUT and pmirGLO-ORF6-MUT respectively. The primers for vector construction are shown in Additional file 1. Marc-145 cells were co-transfected with pmirGLO-CEBPB-WT, pmirGLO-ORF6-WT, pmirGLO-CEBPB-MUT or pmirGLO-ORF6-MUT plasmid and miR-320 mimics, miR-320 inhibitor or negative control using lipofectamine 2000, respectively. Twenty-four hours later, the luciferase activity was measured with PerkinElmer 2030 Multilabel Reader (PerkinElmer, Boston, MA, USA).

### Transfection of siRNA and miRNAs

SiRNA oligos against pig CEBPB (sense 5′-CCUCGCAGGUCAAGAGUAATT-3′), miR-320 mimics (sense 5′-AAAAGCUGGGUUGAGAGGGCGAA-3′) and miR-320 inhibitor (sense 5′-UUCGCCCUCUCAACCCAGCUUUU-3′) were designed and synthesized by GenePharma (GenePharma Shanghai, China). For cell transfection, siRNAs or miRNAs were conducted with Lipofectamine 2000 (Invitrogen, Cashman, CA, USA) as advised by the manufacturer’s protocol. Five hours after transfection of siRNA and miRNA, 3% of the cell maintenance solution was replaced. PRRSV strain WUH3 at MOI of 0.1 was infected with Marc-145 for 1 h and then replaced with standard growth solution, and cells were harvested for 36 h.

### QRT-PCR

Total RNA was extracted from Marc-145 cells or tissues with TRIzol reagent (Invitrogen, Cashman, CA, USA) and reversely transcribed with RevertAid First Strand cDNA Synthesis Kit (Thermo Fisher Scientific, Waltham, MA, USA). The levels of mRNA were detected by quantitative realtime PCR (qRT-PCR). The qRT-PCR reaction was performed in LightCycler 480 II (Roche, Mannheinm, Germany) system. Primers used in qRT-PCR are shown in Additional file 2. U6 or β-actin was applied as the internal control, while the fold changes were calculated using the 2^−ΔΔCt^ method. Absolute quantification was used to detect the PRRSV copy number. The primer ORF7-F/R and ORF7-probe were shown in Additional file 2. The efficiency of PCR reactions was calculated for each primer set by carrying out serial dilutions of a cDNA template and plotting CT values against the log of the template concentrations. The experiments were repeated three times.

### Plasmids construction

To investigate whether miR-320-mediated inhibition of PRRSV replication can be used in therapy, the pre-miR-320 was amplified by PCR with primers miR-320-pF and miR-320-pR using porcine genome DNA as template, the pEGFP-N1-miR-320 plasmids were constructed by inserting the pre-miR-320 into the pEGFP-N1 vector (Clontech, Mountain View, CA, USA) (Figure 4A). The primers for vector construction are shown in Additional file 1.

### Animals

Six, four-week-old piglets were divided into two groups: the pEGFP-N1-miR-320 treatment group and the control group. pEGFP-N1-miR-320 or pEGFP-N1 (2.5 mg/kg of body weight per dose) mixed with D5W solution in a final volume of 3 mL were administered to pigs through intramuscular injection, 1.5 mL of PRRSV strain WUH3 (10^5.2^ TCID_50_) were administered by intramuscular injection after 5 h. The weight, rectal temperature and mental state of piglets were measured twice a day and blood was collected every 3 days. On day 14, we performed pathological dissection and collected all the lungs and PAMs of the pigs.

### Western blot analysis

Marc-145 cells or pig tissues were lysed in RIPA buffer containing 1% (v/v) phenylmethylsulfonyl fluoride (PMSF) (Beyotime, Jiangsu, China). The antibodies and their dilutions were shown as follows, anti-CEBPB (GTX100675, GeneTex, Alton Pkwy Irvine, CA, USA, 1:1000), anti-PRRS virus Nucleocapsid (GTX129270, GeneTex, Alton Pkwy Irvine, CA, USA, 1:1000), anti-β-tubulin (GB11017B, Servicebio, Wuhan, China, 1:1000), anti-β-actin (AC026, ABclonal, Wuhan, China, 1:20 000). The protein expression levels were normalized to corresponding β-actin or β-tubulin. ImageJ software was utilized for the quantitative analysis of Western blot results.

### Histological assay

After being fixed in 4% paraformaldehyde, lung tissues were embedded in paraffin. Lung tissues were analyzed by Hematoxylin–Eosin staining (H&E). The experimental procedures were as previously reported [49]. Finally, these sections were observed under an optical microscope (Olympus BX53, Tokyo, Japan) to detect morphological changes in lung tissue.

### Statistical analysis

All experiments were performed at least three times in triplicate. The differences were assessed using two-tailed t-test or one-way ANOVA. Data were presented as mean ± SD, and *p*-value of < 0.05 and < 0.01 were considered to be significant. All the histograms and graphs were generated with GraphPad Prism version 5.0 and Adobe Photoshop CS5 software, respectively.

## Results

### MiR-320 was differentially expressed in PRRSV-infected/mock-infected PAMs from 4 pig breeds

**Figure 1:**
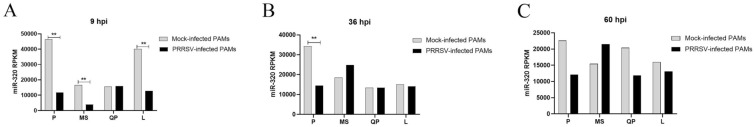
**MiR-320 was differentially expressed in mock vs PRSSV -infected PAMs from 4 pig breeds**. MiR-320 expression levels were analyzed in miRNA-sequencing data of PRRSV-infected/mock-infected PAMs from Pietrain (P), Landrace (L), Qingping (QP), and Meishan (MS) pig breeds at 9 (**A**), 36 (**B**), and 60 (**C**) hpi. Mock-infected PAMs represent PAMs that were infected with culture medium and collected at 9, 36, and 60 h respectively, PRRSV-infected PAMs represent PAMs that were infected with PRRSV and collected at 9, 36, and 60 h respectively. the y-axes on the left present miR-320 RPKM (Reads Per Kilobase per Million mapped reads) expression levels. *p < 0.05, **p < 0.01.

Our previous research on small RNA deep sequencing showed that miR-320 was one of 21 common differentially expressed miRNAs of Meishan, Pietrain, and Landrace pig breeds [22]. The expression level of miR-320 was significantly decreased after 9 h of PRRSV infection in Meishan, Pietrain, and Landrace breeds (Figure 1A), and was not differential in Qingping breed probably caused by low susceptibility to PRRSV. In addition, after 36 and 60 h of PRRSV infection in PAM cells, the expression level of miR-320 showed different trends in the four breeds (Figures 1B and C).

### MiR-320 directly targets PRRSV ORF6

**Figure 2:**
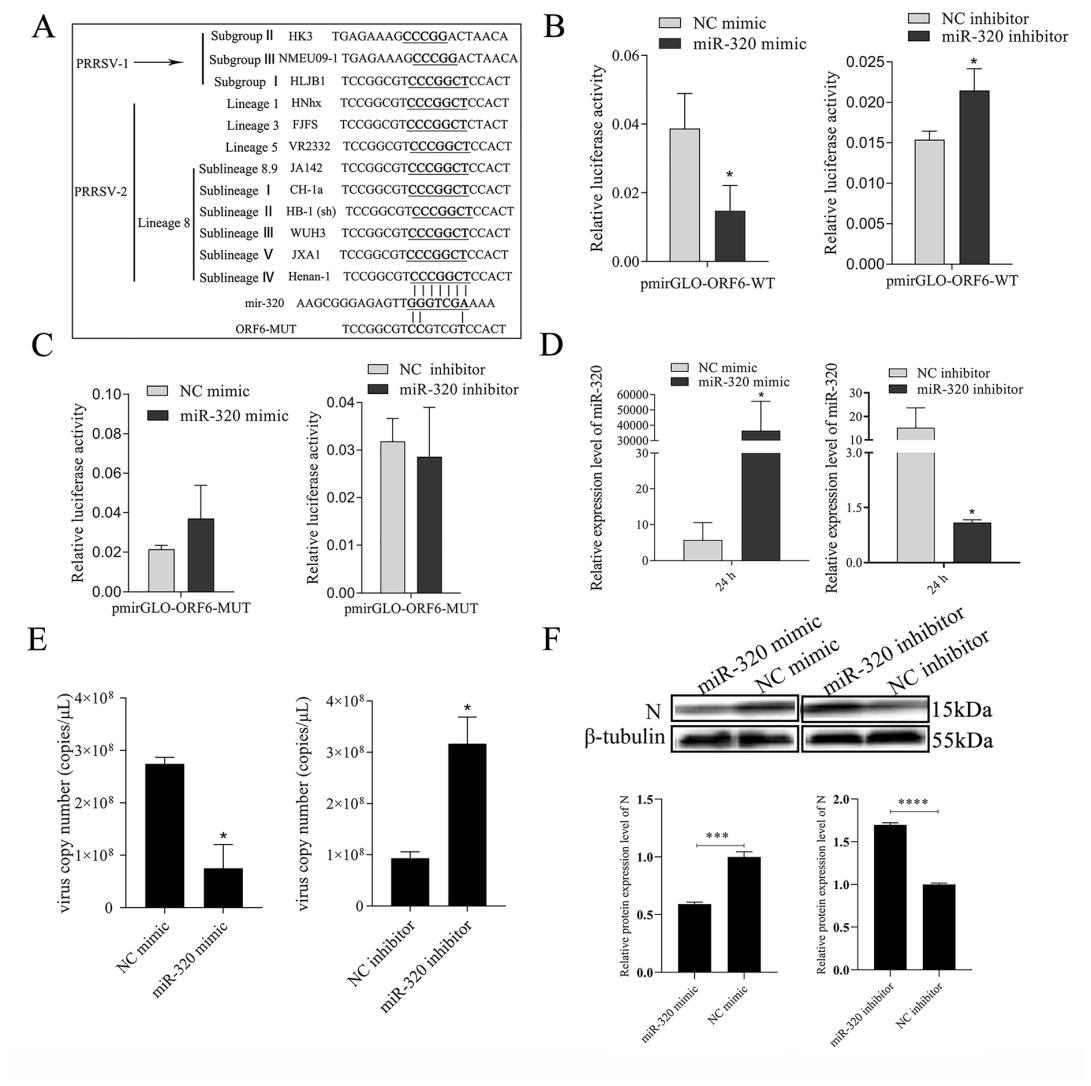
**MiR-320 inhibits the replication of PRRSV. Bioinformatical predication showed that ORF6 was a putative target mRNA of miR-320** (**A**). The dual luciferase reporter assay was used to detect the luciferase activity in cells co transfected with pmirGLO-ORF6-WT/ pmirGLO-ORF6-MUT and miR-320 mimics (**B**) or miR-320 inhibitor (**C**). Marc-145 cells were transfected with miR-320 mimics or miR-320 inhibitor to detect miR-320 expression (**D**). Marc-145 cells were transfected separately with miR-320 mimics and miR-320 inhibitor, and then infected with PRRSV (MOI = 0.1). The cells were harvested at 36 h post PRRSV infection, and qRT-PCR (**E**) and Western blot (**F**) was carried out to detect PRRSV replication. All values represent the mean ± SD of three independent experiments. *p < 0.05.

MicroRNAs have been shown to interact directly with the viral genome to inhibit virus replication. The predicted result by bioinformatics showed that miR-320 could target ORF6 at 14,696 to 14,702 bp of PRRSV genome. In addition, miR-320 could target 9 strains of PRRSV-2 and 1 strain of PRRSV-1 (Figure 2A). Further, it was verified that miR-320 could bind to ORF6 by luciferase reporter analysis. The pmirGLO-ORF6-WT luciferase reporter plasmid was co-transfected with miR-320 mimics or miR-320 inhibitor into Marc-145 cells, and luciferase activity was found to be significantly (*p* < 0.05) suppressed by miR-320 (Figure 2B). However, miR-320 had no effect (*p* > 0.05) on pmirGLO-ORF6-MUT luciferase reporter plasmid (Figure 2C).

### MiR-320 inhibits the replication of PRRSV

To investigate whether miR-320 inhibition of PRRSV replication is caused by binding to ORF6, Marc-145 cells were transfected with miR-320 mimics and miR-320 inhibitor, followed by infection with PRRSV strain WuH3 at an MOI of 0.1cells for 36 h. The absolute quantification results showed that PRRSV copy number was significantly (*p* < 0.05) inhibited by miR-320 mimics and significantly (*p* < 0.05) increased by miR-320 inhibitor in Marc-145 cell (Figure 2E). The Western blot results showed that PRRSV N protein level was significantly reduced by miR-320 mimics. In contrast, the expression of PRRSV N protein was significantly increased by miR-320 inhibitor in Marc-145 cells (Figure 2F). The above-mentioned findings provided evidence that miR-320 could inhibit the PRRSV replication by targeting the genome of PRRSV.

### MiR-320 binding to the CEBPB mRNA

**Figure 3:**
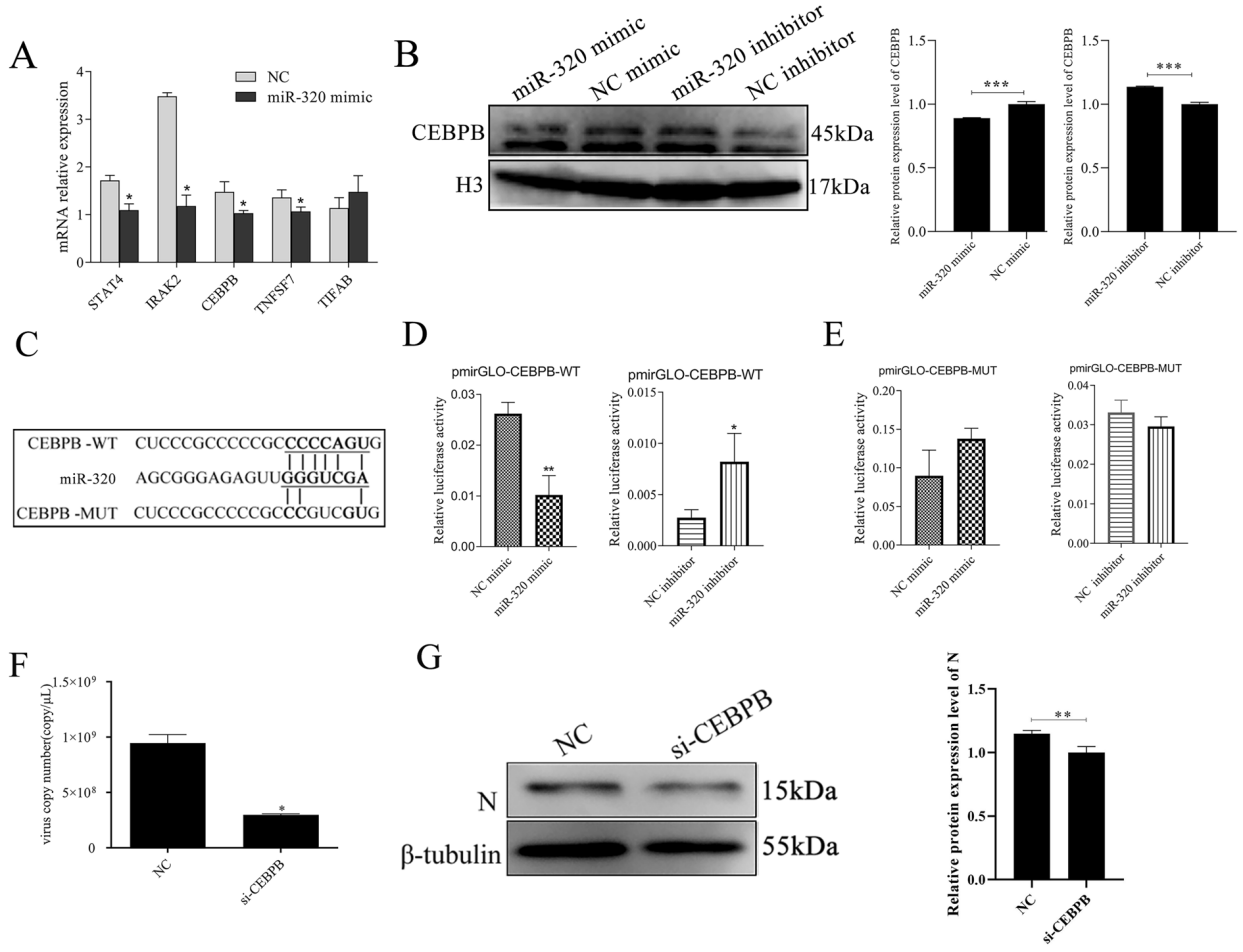
**miR-320 regulate target mRNA CEBPB**. Marc-145 cells were transfected with miR-320 to detect the expression of the potential target mRNAs (**A**). CEBPB protein expression was detected by Western blot after the transfection of Marc-145 cells with miR-320 mimics or miR-320 inhibitor (**B**). The potential target mRNAs predicted by bioinformatics for miR-320 (**C**). The dual luciferase reporter assay was used to detect the luciferase activity in cells co-transfected with miR-320 mimics/inhibitor and pmirGLO-CEBPB-WT (**D**) or pmirGLO-CEBPB-MUT (**E**). Marc-145 cells were transfected with si-CEBPB, and then infected with PRRSV (MOI = 0.1). The cells were harvested at 36 h post PRRSV infection, and qRT-PCR (**F**) and Western blot (**G**) was carried out to detect PRRSV replication. All values represent the mean ± SD of three independent experiments. * p < 0.05, ** p < 0.01, *** p < 0.001.

Host miRNAs were shown to influence the life cycle of RNA viruses by altering host cell gene expression. Using qRT-PCR, 5 predicted potential target mRNAs were examined after miR-320 mimics were transfected into Marc-145 cells. The results showed that STAT4, IRAK2, CEBPB andTNFSF7 were significantly (*p* < 0.05) inhibited by miR-320 mimics (Figure 3A). At the protein level, Western blot analysis also revealed that the expression of CEBPB was significantly inhibited by miR-320 (Figure 3B). To confirm that miR-320 directly targets the 3’UTR of CEBPB, dual-luciferase reporter plasmids carrying the CEBPB 3’UTR with the wild-type or base-pair mutant miR-320 binding regions was constructed (Figure 3C). The results showed that the fluorescent activity of pmirGLO-CEBPB-WT was significantly (*p* < 0.01) inhibited by the miR-320 mimics. The fluorescence activity of pmirGLO-CEBPB-WT was significantly (*p* < 0.01) enhanced when Marc-145 cells were transfected with miR-320 inhibitor (Figure 3D). Neither miR-320 mimics nor miR-320 inhibitor could affect the fluorescence activity of pmirGLO-CEBPB-MUT (Figure 3E). These data demonstrated that miR-320 specifically inhibited CEBPB expression by directly targeting its 3’UTR.

### CEBPB significantly promotes PRRSV replication

In order to elucidate the role of CEBPB in PRRSV infection, Marc-145 cells were transfected with si-CEBPB, followed by infection with PRRSV for 36 h. The results showed that PRRSV replication was significantly (*p* < 0.05) inhibited by interfering with CEBPB (Figure 3F). In addition, at the protein level, the expression of PRRSV N protein was also significantly inhibited when compared to the NC group (Figure 3G). These results showed that CEBPB is necessary for PRRSV replication.

### MiR-320 inhibits PRRSV replication in piglets

**Figure 4:**
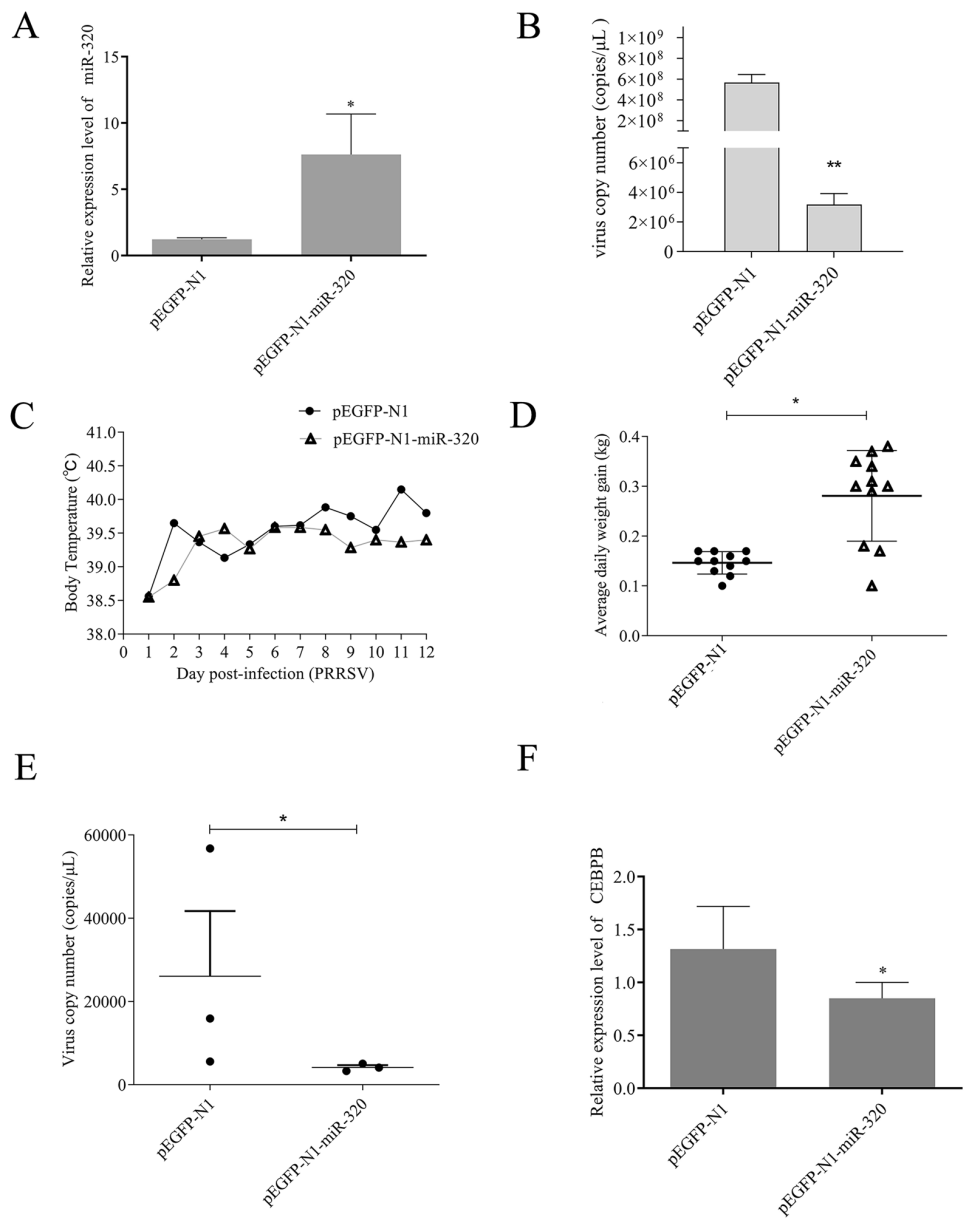
**miR-320 inhibits PRRSV replication in vivo**. Marc-145 cells were transfected with pEGFP-N1-miR-320 or pEGFP-N1 (4 µg) to detect miR-320 expression (**A**) and the virus copy number (**B**). Daily average body temperatures (**C**) and weight gain (**D**) of piglets infected PRRSV after injection with plasmid pEGFP-N1 for the positive control group and pEGFP-N1-miR-320 for the experimental group. Virus copy number (**E**) and CEBPB mRNA expression (**F**) were detected in the lungs of piglets from the experimental group and positive control group. All values represent the mean ± SD of three independent experiments. * p < 0.05, ** p < 0.01.

**Figure 5:**
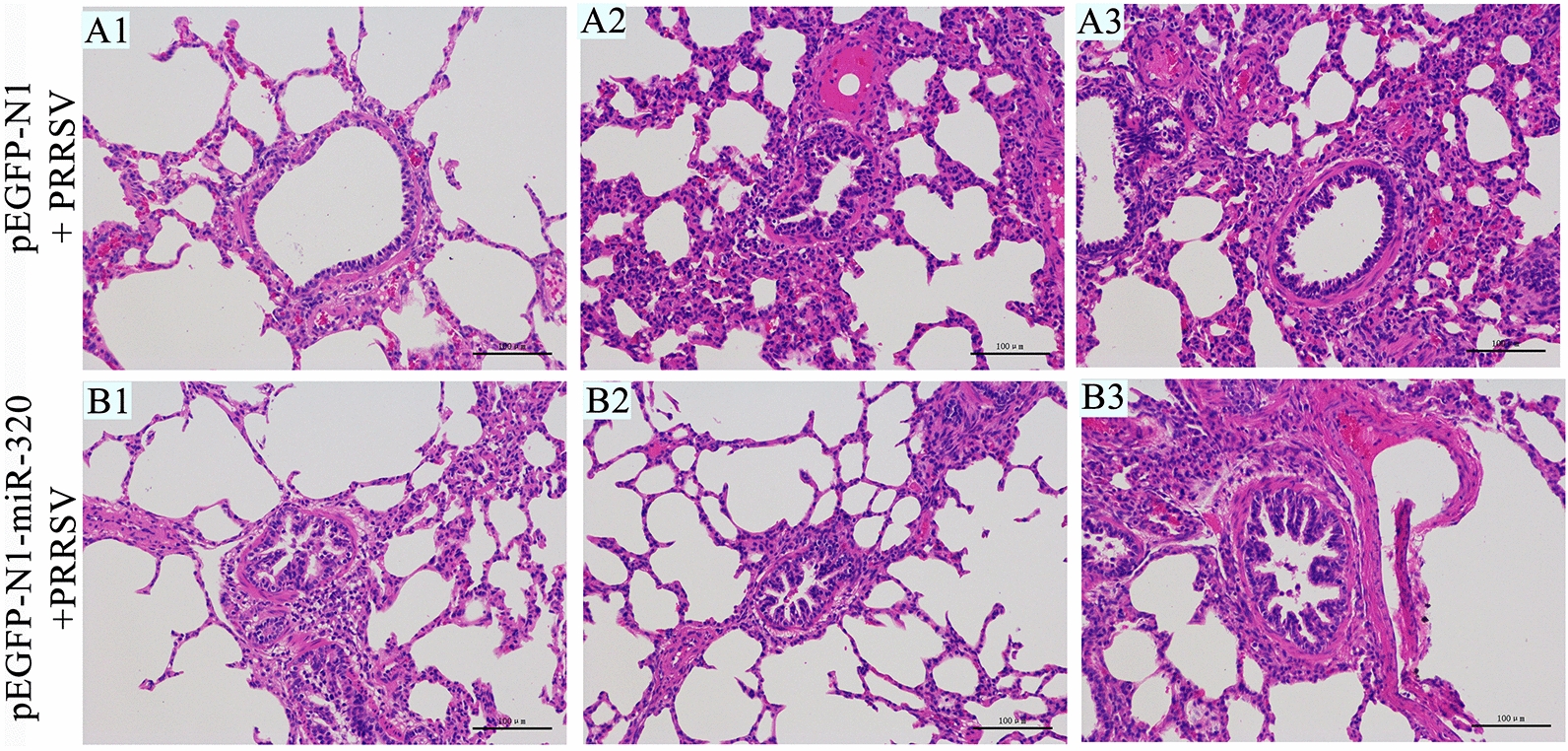
**Histopathological analyse of lungs of piglets from the control and experimental groups in vivo**. Histological sections of lungs of piglets from the experimental group injected with pEGFP-N1-miR-320 and PRRSV infection (B1, B2, B3), the control group injected with pEGFP-N1 and PRRSV infection (A1, A2, A3) were stained with hematoxylin–eosin.

Furthermore, the expression of PRRSV ORF7 in Marc-145 cells was significantly (*p* < 0.01) inhibited by the overexpression of miR-320, transfecting pEGFP-N1-miR-320 (Figure 4B). Subsequently, in 6 four-week-old Landrace piglets, we found that the body temperature of the pigs in thepEGFP-N1 control group increased rapidly and continued to increase after a period of fluctuation. However, the body temperature of the pEGFP-N1-miR-320 experimental group was slowly increased and then maintained at a stable temperature. The results showed that pEGFP-N1-miR-320 could significantly alleviate the fever caused by PRRSV (Figure 4C). At the same time, pEGFP-N1-miR-320 also alleviated the slow growth caused by PRRSV (Figure 4D). Viral load in pig lung tissue was significant reduced in the miR-320 experimental group using qRT-PCR (Figure 4E). The expression of CEBPB in vivo also significantly inhibited by miR-320 (Figure 4F). The lungs of pigs in the pEGFP-N1-miR-320 experimental group showed fewer pathological changes than piglets in the pEGFP-N1 control group (Figure 5). These results showed that miR-320 could inhibit PRRSV replication in vivo.

## Discussion

Many studies have been done on miRNAs and viruses in recent years [23–25]. Previous reports also showed that miRNAs regulated PRRSV replication by directly targeting the PRRSV genome [20, 21]. Li et al. [20] reported that miR-130 family members targeted the 5’UTR of the PRRSV genome and inhibited viral replication. In our study, miR-320 was one of 21 common differentially expressed miRNAs of Meishan, Pietrain, and Landrace pig breeds at 9 hpi. In addition, a previous study found that miR-320a inhibited the infection and replication of enteritis virus in F81 cells by targeting the 3’UTR of transferrin receptor [26]. And miR-320 inhibitor was found to be effective in improving EBOV-induced cytotoxicity [27]. Bioinformatics and experiments found that miR-320 directly targeted PRRSV ORF6, which encoded nonglycosylated membrane protein M [28]. Previous reports have shown that artificial miRNAs significantly inhibited PRRSV replication by targeting PRRSV ORF6 (amirM-82, -217, -263) in Marc-145 cells [29]. Another report showed that the small interfering RNAs of ORF6 could effectively inhibited PRRSV-JXwn06 replication in cultured cells in vitro [30]. It is consistent with our results that miR-320 significantly inhibited PRRSV replication. These results suggest that miR-320 could inhibit replication of PRRSV by targeting ORF6. What is more, the PRRSV GP5 (ORF5 encoded) and M proteins are known to form a heterodimeric complex which is important for viral structure and infectivity [31]. It could be concluded that miR-320 had a significant role in PRRSV infection.

Previous studies have also shown that miRNAs inhibited PRRSV replication by targeting host factors. In this study, we have demonstrated that miR-320 inhibited PRRSV replication by directly targeting PRRSV ORF6. Moreover, the results of bioinformatics analysis and experiment also confirmed that CEBPB was a target mRNA of miR-320. Previous studies showed that CEBPB was essential for efficient HIV replication in macrophages and had been reported to play an important role in regulating HIV/SIV replication in alveolar macrophages [32–34]. Other studies showed that CEBPB^−/−^ mice were found to be resistant to oral candidiasis, showing increased susceptibility only under conditions of steroid-induced immunosuppression [35]. Previous studies had shown that increased miR-155 level could help to reduce HBV load by targeting CEBPB in vitro [36]. Consistent with earlier reports, the knockdown of CEBPB by siRNA inhibited the replication of PRRSV. This result demonstrated that CEBPB significantly promoted the replication of PRRSV. Our studies also showed that overexpression of miR-320 in Marc-145 cells significantly down-regulated the expression of both CEBPB mRNA and protein. These results indicated that miR-320 also regulated PRRSV replication via CEBPB.

Prevention and control of many livestock and poultry viral infections can no longer rely on traditional vaccines such as inactivated and attenuated vaccines [37, 38]. The emergence of DNA vaccines has improved this situation [39, 40]. Plasmid DNA encoding different antigenic genes, can cause substantial and long-lasting immune responses in many species of vertebrates such as mammals, birds and fish. Meng cloned the GP5 gene into a eukaryotic expression plasmid under the control of the cytomegalovirus early promoter to prepare a DNA vaccine, which could induce the production of antibodies after immunization of piglets [41]. More and more studies showed that miRNAs could be used to treat PRRS [42–44]. In our research, intramuscular injection of pEGFP-N1-miR-320 could reduce the replication of PRRSV in pigs. In addition, miR-320 could effectively alleviate the symptoms caused by PRRSV in vivo. The average weight gain was particularly significant. The pigs in the control slightly increased their body weight after infection with the virus, while the weight gain of the miR-320 treatment piglets was substantial. HE staining experiments showed that miR-320 could protect the piglets from damage. The interstitial pneumonia was more severe in the lungs of the control group than that of the experiment group. The interstitial enlargement and congestion were more prominent in the control group than that in the experiment group.

In summary, the present study demonstrated that miR-320 bound to the CEBPB 3'UTR by seed sequence pairing to regulate CEBPB expression and targeted ORF6 of PRRSV, thereby inhibiting PRRSV replication. In addition, miR-320 could effectively alleviate the symptoms caused by PRRSV in vivo. It could be concluded that miR-320 played an essential role during PRRSV infection. These insights may be applicable to PRRS prevention and control.

In summary, we discovered that miR-320 significantly inhibited PRRSV replication by suppressing the expression of CEBPB and directly targeting PRRSV ORF6. These data suggest that miR-320 has significant roles in the infection and may be promising therapeutic target for PRRS.

### Supplementary Information

**Additional file 1.** **Sequences of primers used for plasmid construction in this study**.

**Additional file 2.** **Quantitative RT-PCR primer sequences used in this study**.

## Data Availability

All data generated or analyzed during this study are included in this published article and its supplementary information files.
