# Supplementary material for: MiR-320 inhibits PRRSV replication by targeting PRRSV ORF6 and porcine CEBPB
Source: Vet Res. 2024 May 15;55:61. doi: 10.1186/s13567-024-01309-7 (PMC11097481; doi:10.1186/s13567-024-01309-7)
Supplement: Supplementary file 1 — Additional file 1. Sequences of primers used for plasmid construction in this study. [file 13567_2024_1309_MOESM1_ESM.docx]

# Additional file 1 Sequences of primers used for plasmid construction in this study.

| Primers’ name | Sequences of primers |
| --- | --- |
| CEBPB-W-F | CCGCTCGAGCGGCTGGAGACGCAGCATAAGG |
| CEBPB-W-R | GCTCTAGAGCTGTGCGGTTGGTTTGGA |
| ORF6-W-F | CGAGCTCGTACTTACACGCCAGTGATGAT |
| ORF6-W-R | CCGCTCGAGCGGTTCTGCCACCCAACACG |
| CEBPB-M-R | GCTCTAGACAAAGTTTTGCCGGCGCCA*GACC*GG |
| CEBPB-M-F | CCGCTCGAGCC*GGTC*TGGCGCCGGCAAAACTTTG |
| ORF6-M-R | CCGCTCGAGTGGA*CGAC*GGACGCCGGACGACAAA |
| ORF6-M-F | CGAGCTCTTTGTCGTCCGGCGTCC*GTCG*TCCA |
| miR-320-pF | CCGCTCGAGCGGTAACTTCACATCCAATGCGACC |
| miR-320-pR | GGAATTCCAGCAGCGAATCCCCACA |
