# Supplementary material for: MiR-320 inhibits PRRSV replication by targeting PRRSV ORF6 and porcine CEBPB
Source: Vet Res. 2024 May 15;55:61. doi: 10.1186/s13567-024-01309-7 (PMC11097481; doi:10.1186/s13567-024-01309-7)
Supplement: Supplementary file 2 — Additional file 2. Quantitative RT-PCR primer sequences used in this study. [file 13567_2024_1309_MOESM2_ESM.docx]

# Additional file 2 Quantitative RT-PCR primer sequences used in this study.

| **Primers’ name** | **Sequences of primers** |
| --- | --- |
| miR-320-loop | CTCAACTGGTGTCGTGGAGTCGGCAATTCAGTTGAGTTCGCCCT |
| miR-320-F | ACACTCCAGCTGGGAAAAGCTGGGTTGAG |
| Universal primer-R | TCAACTGGTGTCGTGGAGTCGGC |
| U6-F | GCTTCGGCAGCACATATACT |
| U6-R | TTCACGAATTTGCGTGTCAT |
| β-actin-F  β-actin-R | CCAGGTCATCACCATCGG  CCGTGTTGGCGTAGAGGT |
| ORF7-F | TCAGCTGTGCCAAATGCTGG |
| ORF7-F | AAATGGGGCTTCTCCGGGTTTTT |
| Probe-ORF7 | TCCCGGTCCCTTGCCTCTGGA |
| GAPDH-F | TCATGACCACAGTCCATGCC |
| GAPDH-R | GGATGACCTTGCCCACAGCC |
| STAT4-F | CCTTTGGATTGATGGGTATG |
| STAT4-R | GTCAGCGAATGGTAGAGCAG |
| IRAK2-F | CAGACTCCCTGGATGTTGTG |
| IRAK2-R | GAGGTGCTGACTTCCTTTGA |
| TIFAB-F | GCCTTTGTCTGCTTCTTCCAT |
| TIFAB-R | GCTGTATTTCTGTTGCTCCTCC |
| CEBPB-F | TGTCCACATCCTCGTCGTCC |
| CEBPB-R | TGCTGCGTCTCCAGGTTGC |
| TNFSF7-F  TNFSF7-R | CACCACCCACAGCATTAGC  GAAGGCAGCAGAGGCAGA |
